# Conditional trust pathways in live-streaming commerce: how consumer motivation influences responses to human and AI anchors

**DOI:** 10.3389/fpsyg.2026.1797803

**Published:** 2026-05-04

**Authors:** Yujia Lin, Yue Zou, Xin Wang

**Affiliations:** 1 School of Journalism and Communication, Sichuan International Studies University, Chongqing, China; 2 School of Communication, Fujian Normal University, Fuzhou, Fujian, China; 3 School of Journalism and Communication, Chongqing University, Chongqing, China

**Keywords:** anchors, human–AI interaction, live-streaming commerce, motivational relevance, online identity perception, trust formation

## Abstract

**Background:**

AI anchors are increasingly deployed in live-streaming commerce, raising the question of whether they can substitute for human anchors. Prior studies have documented differences in consumer responses to these anchor types, but the psychological processes underlying trust formation remain unclear. This study approaches the question from a media psychology and human-machine communication perspective rather than focusing solely on commercial outcomes.

**Methods:**

A between-subjects experimental design was employed. Participants (*N* = 439) were randomly assigned to watch a live-streaming sales video hosted by either a human anchor or an AI anchor. Participants then completed measures of perceived intimacy, perceived responsiveness, trust, purchase intention, and motivational orientations (hedonic and utilitarian). A six-factor confirmatory factor analysis confirmed the measurement model, and moderated mediation analyses were conducted with heteroscedasticity-consistent standard errors.

**Results:**

Human anchors generated higher trust and purchase intention overall. Anchor type influenced trust through two asymmetric identity-based cue pathways. Perceived intimacy (a relational cue) mediated the effect of anchor type on trust, particularly when hedonic motivation was moderate to high. Perceived responsiveness (a functional cue) did not function as a general mediator; it became a significant pathway favoring AI anchors only when utilitarian motivation was high. At low utilitarian motivation, this pathway reversed direction.

**Conclusion:**

Consumer trust in live-streaming commerce is a conditional, motivation-dependent process rather than a uniform preference for either anchor type. Human anchors build trust through a broadly effective relational pathway, while AI anchors’ functional advantage converts into trust only under specific motivational conditions. These findings suggest that AI anchors will not broadly replace human anchors, but can be strategically effective when matched to efficiency-oriented consumer goals.

## 1 Introduction

The continuous evolution of social media platforms has expanded the ways in which individuals access information and engage in interaction, leading to a rapid increase in consumers’ demand for real-time communication (Xie et al., 2024). As an important functional form embedded within social media platforms, live-streaming commerce has gained widespread popularity among online consumers due to its immediacy and interactivity (Li G. et al., 2023: Li S. et al., 2023). Live-streaming commerce refers to a commercial model in which anchors share product trials and usage experiences through live-streaming sessions, providing consumers with product demonstrations and immediate purchasing opportunities (Hu and Chaudhry, 2020). This model integrates real-time video presentation, interactive communication, and instant purchasing within a single communicative context. During live streams, anchors can convey not only the functional value of products (Jiao et al., 2023) but also their emotional value, thereby enhancing consumers’ purchase intentions. Compared with traditional e-commerce, live-streaming commerce places anchors at the core of the consumption experience. Through real-time, vivid, diverse, and highly interactive presentations, anchors organize product information, sustain audience attention, and stimulate purchase intention, ultimately facilitating the conversion from live viewing to product purchase (Jiao et al., 2023; Wongkitrungrueng et al., 2020; Xue and Liu, 2022). In this process, anchors not only present product information but also continuously shape consumers’ cognitive interpretations, emotional responses, and decision tendencies through interactive discourse.

In recent years, advances in artificial intelligence technologies have accelerated the adoption of AI virtual anchors in live-streaming commerce. AI anchors are defined as digital virtual agents that employ big data, virtual synthesis technologies, and human–computer interaction (HCI) systems to autonomously perform tasks traditionally undertaken by human anchors to a certain extent (Wang and Zhu, 2022; Zhang et al., 2024). Prior research distinguishes between AI-powered virtual anchors and human-powered virtual anchors (Wang et al., 2024). Due to early technological constraints, AI-powered virtual anchors were limited in their real-time interactivity and situational adaptability, making it difficult for them to respond promptly to consumer needs; as a result, their live-streaming performance often failed to meet brand expectations (Belanche et al., 2024). Consequently, existing research has primarily focused on differences between human anchors and human-powered virtual anchors (Xie et al., 2024). Within this line of inquiry, scholars have noted that live-streaming scenarios involving AI virtual anchors tend to exhibit strong virtual influence characteristics, which may suppress emotional integration processes and hinder the development of consumer empathy (Wang et al., 2024).

Nevertheless, with ongoing technological advancements, the latest generation of AI-powered virtual anchors, centered on virtual avatars, synthetic voices, and algorithm-driven content generation, has become increasingly capable of simulating human anchors’ presentation styles and interaction processes in live-streaming contexts. As AI anchors become increasingly human-like, technological distinctions alone are insufficient to explain consumer responses. In particular, in live-streaming commerce, where uncertainty and time pressure are salient, identity-based interpretations are likely to translate into trust judgments. As a result, a growing number of brands have begun to experiment with AI-powered virtual anchors in live-streaming commerce (Hu and Ma, 2023; Zhang et al., 2024). Importantly, AI-powered virtual anchors are not generated entirely independently of human characteristics. Rather, they often learn from and replicate human anchors’ expressive patterns in terms of tone, facial expressions, and hosting scripts, and in some cases are developed as digital doubles of real human anchors’. For example, the AI agent of the well-known Chinese internet celebrity Luo Yonghao achieved a total transaction volume exceeding 55 million RMB in its first live-streaming session, attracting more than 13 million viewers. This type of AI anchor demonstrates several practical advantages, including lower operational costs, the ability to conduct live streams continuously around the clock, and freedom from temporal constraints. Moreover, because their behaviors and expressions are governed by pre-programmed AI systems, such anchors can reduce certain risks inherent in live-streaming commerce (Wu et al., 2023). In this context, consumers increasingly evaluate AI anchors that closely resemble human anchors as interactive partners, raising a main research question: how do consumers perceive differences in online identity construction across different types of anchors? Understanding these perceived identity differences is critical, as they may fundamentally shape how consumers evaluate credibility and form trust in live-streaming commerce.

Unfortunately, prior research has devoted substantially more attention to the online identity construction of human anchors than to that of AI anchors. Existing live-streaming commerce studies predominantly focus on human anchors, emphasizing characteristics such as credibility, expertise, friendliness, warmth, and attractiveness, and consistently find that these attributes enhance consumers’ purchase intentions (Gao et al., 2023; Wang et al., 2024; Wongkitrungrueng et al., 2020). In contrast, the limited body of research addressing AI anchors has largely concentrated on human-powered virtual anchors rather than AI-powered virtual anchors, and has remained largely descriptive in nature (Audrezet and Koles, 2023; Belanche et al., 2024). In addition, prior studies suggest that AI anchors have difficulty forming independent product evaluations and tend to exhibit weaker social affinity (Arsenyan and Mirowska, 2021). At the same time, because AI anchors are often designed by learning from and mimicking the expressive styles and behavioral patterns of human influencers, some studies have inferred that their persuasive effects may be comparable to those of human anchors (Sands et al., 2022; Stein et al., 2024).

Behind these contradictory findings lies a question of considerable practical and theoretical significance: can AI anchors, which are capable of operating around the clock at lower cost, broadly replace human anchors in live-streaming commerce? This question has produced a deadlocked debate. On one side, AI anchors offer scalability and behavioral consistency; on the other, human anchors offer emotional spontaneity, relational depth, and social adaptability. Yet this debate has been conducted largely in terms of commercial outcomes, asking which anchor type generates more sales or higher engagement. What has received far less attention is the underlying psychological question: how do consumers, as media users, psychologically process AI versus human communicators in an interactive media environment? The replacement debate, we argue, is at its core a question about human–machine communication and media psychology, not merely one of marketing efficiency.

Research in the psychology of AI provides a useful starting point. A growing body of work has documented “algorithm aversion,” a general tendency for individuals to exhibit reluctance or resistance toward algorithmic agents compared to human agents, manifesting in more negative emotional reactions, less favorable perceptions, and lower willingness to rely on algorithms (Williams and Lim, 2024). This aversion has been attributed to several psychological mechanisms, including the perceived inability of algorithms to account for unique, qualitative, or subjective information, and the belief that algorithms lack the capacity for genuine emotional experience (Williams and Lim, 2024). Such findings suggest that consumer responses to AI anchors are not simply a function of AI performance, but are shaped by deeper psychological inferences about the nature and identity of the communicative agent. This perspective shifts the analytical lens from “what AI does” to “what consumers believe AI is” when they encounter it in a mediated interaction.

At the heart of the replacement debate lies the issue of trust. Trust plays a critical role in human–machine communication, as it is essential for establishing effective and reliable interactions between humans and technological agents (Omrani et al., 2022). In live-streaming commerce specifically, purchasing decisions are made under conditions of information asymmetry, heightened time pressure, and limited opportunity for independent product verification. Anchors may further intensify perceived urgency through tactics such as limited-time offers or low-stock cues, increasing consumers’ reliance on anchors as both information sources and risk buffers. Under such conditions, whether consumers trust the anchor becomes the critical psychological mechanism determining whether viewing converts into purchase. Yet existing research on trust in AI systems has largely examined institutional or service contexts (Omrani et al., 2022), while live-streaming commerce research has focused on trust as an outcome of human anchors’ characteristics (Liu et al., 2025; Wongkitrungrueng and Assarut, 2020). The question of how trust forms differently when the communicative agent is AI versus human, within an interactive media environment, remains underexplored.

A central limitation of existing research is that it has approached this question from a predominantly business perspective, documenting observable differences in consumer outcomes without explaining the underlying psychological processes. Studies have compared AI and human anchors on purchase intention, engagement, or satisfaction (Gao et al., 2025; Liu et al., 2025), but have not provided a mechanism-based account of why consumers respond differently to these two types of agents. From a media psychology perspective, what is needed is not merely a comparison of outcomes, but an analysis of the psychological cue pathways through which different types of communicators build (or fail to build) trust in the minds of their audiences. Specifically, we need to understand: which identity cues do consumers attend to when evaluating AI versus human anchors? And under what conditions do these cues translate into trust?

The present study addresses these questions by identifying two categories of identity-based cues that are particularly salient in the live-streaming interaction context. The first category consists of relational cues, which signal closeness, benevolence, and social alignment. We operationalize relational cues as perceived intimacy, reflecting consumers’ felt emotional closeness and parasocial connection with the anchor. In mediated interactions, such cues are often manifested through reduced emotional distance and enhanced feelings of interpersonal proximity. The second category consists of functional cues, which signal competence, attentiveness, and task execution ability. We operationalize functional cues as perceived responsiveness, referring to the extent to which an anchor is perceived as providing timely and effective responses to consumer needs. These two constructs capture how consumers evaluate the communicative agent along the fundamental dimensions of social judgment that have been identified in prior work on streamer characteristics (Gao et al., 2025). Importantly, while prior research has examined relational and functional attributes of live-streaming anchors separately (Liao et al., 2022; Gao et al., 2023), no study has integrated them into a unified framework that compares how these cue pathways operate differently for AI and human anchors in building trust.

We further propose that whether a given identity cue translates into trust depends on consumers’ motivational orientations. Consumers do not enter live-streaming shopping contexts as passive recipients of information. They participate with different goals. Hedonic motivation emphasizes experience, emotion, and entertainment value. Utilitarian motivation emphasizes efficiency, practicality, and problem solving. Prior research has shown that these motivational orientations influence how consumers emotionally respond to anchors, with consumers holding different shopping values exhibiting different emotional and evaluative patterns (Liu et al., 2025). We extend this insight by proposing that hedonic and utilitarian motivations function not merely as predictors of behavior, but as boundary conditions that determine which type of identity cue carries greater weight in trust formation. When hedonic motivation is salient, consumers may rely more on relational cues, strengthening the link between perceived intimacy and trust. When utilitarian motivation is salient, consumers may rely more on functional cues, strengthening the link between perceived responsiveness and trust. This motivation-dependent cue-weighting process, if supported, would reframe the replacement debate: AI anchors are not generally inferior to human anchors, nor are they generally equivalent. Their effectiveness is conditional on the alignment between the cues they project and the goals consumers bring to the interaction.

To test these propositions, this study proposes a human–AI agent trust formation framework for live-streaming commerce. The framework specifies two mediating pathways from anchor type to trust: a relational pathway grounded in perceived intimacy and a functional pathway grounded in perceived responsiveness. Hedonic and utilitarian motivations moderate the strength of these pathways. Trust is conceptualized as a proximal antecedent of purchase intention, linking psychological evaluation to behavioral outcome.

This study makes three contributions. First, it reframes the AI replacement debate as a question about human–machine communication and media psychology rather than commercial performance, proposing that the relevant question is not “which anchor is better” but “through which psychological pathways does each type of communicator build trust, and under what motivational conditions.” Second, by identifying perceived intimacy and perceived responsiveness as distinct mediators with different boundary conditions, the study offers a mechanism-based account that can explain inconsistent findings in prior research: AI anchors may appear ineffective in studies that do not account for consumers’ motivational states, but may perform comparably or even favorably among efficiency-oriented consumers. Third, by introducing hedonic and utilitarian motivations as moderators of the cue-to-trust link (rather than merely as predictors of behavior), the study proposes a novel motivation-dependent trust model that may generalize to other contexts where human and AI communicative agents coexist in mediated environments. At the practical level, the framework provides implications for anchor selection and AI design. If the two anchor types build trust through different cue pathways, then the optimal choice depends on the product category, the target consumer segment, and the motivational context of the viewing occasion. Furthermore, the findings can inform AI anchor design by suggesting that platforms should calibrate their AI systems to the relevant cue pathway rather than pursuing blanket anthropomorphism.

## 2 Theoretical background and hypothesis development

### 2.1 AI and human anchors

Live-streaming commerce is an interactive form of social commerce that integrates video streaming with entertainment formats such as talk shows and real-time chatting (Robertson, 2022). Compared with traditional social commerce, live-streaming commerce differs in at least three ways (Liao et al., 2022). First, in conventional online shopping, consumers primarily rely on pictures and text to learn about products. By contrast, live-streaming commerce provides real-time video demonstrations, which allow viewers to obtain richer and more comprehensive product information (Wongkitrungrueng and Assarut, 2020). Second, live shopping enables viewers to ask questions via live chat, and anchors can respond promptly (Wongkitrungrueng and Assarut, 2020). Third, in traditional social commerce, the lack of face-to-face communication may increase viewers’ doubts about seller credibility, thereby elevating perceived transaction risk (Liao et al., 2022). In live-streaming contexts, however, anchors interact with viewers in real time and sustain dialogue throughout the session (Liao et al., 2022). When shopping is embedded in a conversational format, viewers are more likely to feel that their opinions are heard and taken seriously by the anchors (Zhang S. et al., 2022; Zhang M. et al., 2022). Such immediate two-way communication can help anchors build emotional connections with viewers, enhance enjoyment, and ultimately trigger purchase behaviors (Hu and Ma, 2023; Hu and Chaudhry, 2020; Wang et al., 2022).

Prior research has identified three broad categories of factors associated with anchor success in this context (Hu and Ma, 2023; Zheng et al., 2023). The first category concerns the anchor’s image, such as physical attractiveness (Go and Sundar, 2019). The second concerns personal style, including expertise, interactivity, humor, enthusiasm, friendliness, and response speed (Zhang S. et al., 2022; Zhang M. et al., 2022; Zheng et al., 2023). The third concerns language use, such as literal versus metaphorical expression (Leung, 2021) and emotional versus rational appeals (Ahn et al., 2022). Across these dimensions, human anchors may be more readily recognized as socially skilled communicators, because they can draw on a wider range of socially grounded cues such as humor and context-sensitive storytelling. Human anchors may also be more capable of building a stronger sense of social presence through interactivity, emotional support, and community feelings (Chen and Liao, 2022). Social presence can enhance users’ sense of “being there” by creating vicarious experiences and social interaction (Hu et al., 2017), which may further facilitate intimacy, warmth, and enjoyment (Ma, 2021), thereby promoting purchase intention.

A critical question is whether these persuasive and interactive advantages, which have been documented primarily for human anchors, remain valid when the communicative agent transitions from a human to an AI agent. From a media psychology perspective, this question concerns how audiences psychologically process different types of communicators in a mediated environment. The Computers Are Social Actors (CASA) paradigm demonstrates that individuals tend to apply social rules and expectations to technological agents, even when aware of their non-human nature (Nass and Moon, 2000; Reeves and Nass, 1996). This suggests that AI anchors may indeed be processed as social agents by viewers. However, the CASA paradigm also implies that the social inferences viewers draw may differ in content depending on the cues that the agent projects. A growing body of research in the psychology of AI has documented “algorithm aversion,” a general tendency for individuals to exhibit reluctance toward algorithmic agents, manifesting in more negative emotional reactions, less favorable perceptions, and lower willingness to rely on algorithms (Williams and Lim, 2024). This suggests that even when AI anchors are perceived as social agents, the nature of the social judgments formed about them may differ from those formed about human anchors. We therefore expect that, at an overall level, human anchors will generate more favorable consumer responses. Based on this reasoning, we propose the following hypothesis:

*H1*: Human anchors are expected to generate higher purchase intention than AI anchors at an overall level.

### 2.2 Trust

Due to earlier technological constraints, research adopting a psychological mechanism perspective remains limited regarding how newly emerging AI anchors and human anchors shape viewers’ purchase-related outcomes. Prior studies have largely focused on the direct effects of live streaming on purchase intention (Liao et al., 2022), with only a smaller body of work examining motivations, values, and psychological antecedents (Wongkitrungrueng and Assarut, 2020). Against this backdrop, trust can be conceptualized as a pivotal anticipatory evaluation that viewers form after being influenced by anchors (Liu et al., 2025).

Trust, as a psychological state, refers to a positive expectation regarding another party’s intentions or behaviors (Rousseau et al., 1998). The experience of interpersonal trust results from a series of psychological processes shaped by the interplay of personal values, attitudes, emotions, and perceived charisma (Liu et al., 2025). Trust plays a critical role in human–machine communication, as it is essential for establishing effective and reliable interactions between humans and technological agents (Omrani et al., 2022). Prior research has suggested that trust may serve as a key trigger for purchase behavior (Park and Lin, 2020; Wongkitrungrueng et al., 2020; Wongkitrungrueng and Assarut, 2020; Zhang S. et al., 2022; Zhang M. et al., 2022). In live-streaming commerce, anchors can build trust through product introductions and recommendations, thereby reducing product uncertainty (Wongkitrungrueng et al., 2020). Trust reflects not only confidence in the anchor’s expertise, but also beliefs about product safety and reliability and judgments about information credibility; this process depends on consumers’ perceptions of external stimuli and affective interaction (Meng et al., 2021). Once viewers develop trust in an anchor, they are more likely to perceive the anchor as honest and benevolent. Viewers may then map the anchor’s verbal descriptions onto their internal assessment of the product’s true quality and further infer that the product fits their personal needs and preferences (Benbasat and Wang, 2005; Qiu and Benbasat, 2005). Consequently, trust reduces perceived risk, encourages participation, and increases purchase intention (Hock et al., 2010; Liu et al., 2025; Lu and Chen, 2021). We hypothesize:

*H2*: Trust in the anchor positively influences consumers’ purchase intention.

Importantly, it is critical to understand how different types of anchors cultivate trust. AI anchors are constructed by drawing on human internal and external characteristics, with expertise and attractiveness serving as fundamental reference points for imitation (Guo et al., 2022; Liao et al., 2022). Although anthropomorphism has been identified as a key factor shaping consumers’ trust in AI virtual anchors, such that more anthropomorphic AI anchors are more readily accepted and engaged with Chen et al. (2024), current technology may still fall short on several social elements that are consequential for trust formation, including perceived emotional investment, relational intent, and context-sensitive judgment. Thus, while anthropomorphism represents technological progress, whether it can substitute for the socio-psychological foundations through which human anchors elicit trust remains an open question. For example, anchors with higher social status are found to gain greater consumer trust (Wei and Xi, 2023), and viewers tend to trust anchors with stronger reputations (Jiang et al., 2022). Human anchors also frequently employ economic incentives such as lotteries and coupons to stimulate participation, which may further deepen the affective bond between anchors and viewers (Johnson and Woodcock, 2019). By contrast, AI anchors may not possess the same flexibility and may be less able to deliver spontaneous social responses based on real-time interaction dynamics. Without the capacity for genuine emotional reciprocity, AI anchors may face a relative disadvantage in trust formation. Accordingly, we hypothesize:

*H3*: Anchor type influences consumer trust, such that human anchors tend to elicit higher trust than AI anchors.

### 2.3 Identity cues

Social cognitive theory posits that individual behavior is shaped by cognitive processes and is partially determined by how people interpret social information (Bandura, 1986). In the context of live-streaming commerce, consumers evaluate anchors along two fundamental dimensions of social judgment: warmth and competence (Aaker et al., 2010; Gao et al., 2025). Warmth reflects perceptions of benevolence, friendliness, and sincerity, whereas competence reflects perceptions of efficiency, expertise, and skill (Gao et al., 2025). As a central component of warmth, intimacy constitutes the foundation of the relational bond between consumers and anchors (Sousa et al., 2010). As a core component of competence, responsiveness reflects the anchor’s ability to meet consumer needs effectively (Xu and Liu, 2022).

In live-streaming settings, identity cues refer to observable expressive and responsive features during interaction that audiences use to infer an anchor’s intentions and capabilities. To operationalize this judgment framework, the present study conceptualizes relational cues as perceived intimacy and functional cues as perceived responsiveness. It should be noted that perceived intimacy and perceived responsiveness represent key manifestations of relational and functional cues, respectively, but do not exhaust the full conceptual scope of warmth and competence. We focus on these two constructs because they are the most directly observable identity cues in a live-streaming interaction: intimacy is inferred from the anchor’s emotional expressiveness, approachability, and parasocial behaviors, while responsiveness is inferred from the anchor’s speed, attentiveness, and information delivery. Prior research suggests that perceived intimacy and perceived responsiveness may mediate the process through which consumers form trust (Xu and Liu, 2022; Gao et al., 2025).

Perceived intimacy refers to the emotional closeness and sense of personal connection that consumers experience during interactions with an anchor, reflecting the depth of the consumer–anchor relationship (Gao et al., 2025). Such interaction can be understood as a form of parasocial interaction, defined as a perceived relationship between audiences and mediated personas (Horton and Wohl, 1956). Parasocial interaction can partially substitute for face-to-face relationships and enable audiences to derive feelings of affiliation and affection through mediated encounters (Lee and Lee, 2022). Previous studies have shown that intimacy mediates the relationship between cultural factors and purchase intention in social commerce (Yin et al., 2019); that perceived intimacy has a direct positive effect on purchase intention in YouTube contexts (Lee and Lee, 2022); and that anchors’ interaction-oriented behaviors enhance immersion and parasocial interaction, which in turn increase purchase intention (Liao et al., 2022). Through parasocial interaction experiences, audiences may come to perceive anchors as “close friends” and develop emotional bonds that reduce uncertainty and consumption-related concerns, thereby strengthening trust (Nagel et al., 2021). However, the effects of different types of communicative agents may vary. Research in the psychology of AI indicates that individuals exhibit more negative emotional reactions to algorithmic agents and perceive them as less capable of genuine emotional experience (Williams and Lim, 2024). Consumers also tend to accept human service providers more readily and perceive their communication quality as superior to that of AI systems (Song et al., 2022). Human anchors may therefore be better able to establish intimate and personalized relationships through service interactions (Li G. et al., 2023; Li S. et al., 2023). Consequently, AI anchors may be less capable than human anchors of satisfying consumers’ preferences for perceived intimacy, leading to differences in trust formation. Based on this reasoning, we propose:

*H4*: Perceived intimacy mediates the relationship between anchor type and trust, such that human anchors elicit higher perceived intimacy than AI anchors.

Perceived responsiveness refers to consumers’ ability to obtain timely and effective responses from an anchor (Xue et al., 2020). It reflects the extent to which consumers believe that an anchor can address their needs promptly and flexibly, and it constitutes a key element of service quality and consumer engagement (Gao et al., 2025). Prior research shows that response speed influences online purchase behavior (Khoi and Le, 2024), and that AI anchors’ responsiveness can directly and indirectly affect purchase intention (Gao et al., 2023). Compared with AI anchors, human anchors typically rely on directors and personal attention to identify audience messages, whereas AI anchors primarily depend on algorithms and data processing. The former involves experiential judgment, which may yield higher interpretive accuracy but can be subject to delays, while the latter relies on automated processing, which may offer speed advantages but can suffer from contextual mismatches. Previous studies indicate that AI systems may produce ineffective responses, information bias, or lack empathy (Yang et al., 2023), and that AI anchors may be less capable than human anchors of providing contextually appropriate responses (Gao et al., 2025). Responsiveness thus encompasses not only speed but also the appropriateness and effectiveness of responses, and different anchor types may exhibit distinct trade-offs between these dimensions.

However, technological advances may be altering this balance. AI anchors, through algorithmic training and memory learning, may already outperform human anchors in addressing common, standardized inquiries (e.g., pricing or delivery information), and may achieve more stable point-to-point responsiveness. In contrast, human anchors must manage overall stream dynamics and multitasking, which may limit their ability to respond individually when audience volume is high. Moreover, human anchors’ performance may fluctuate due to fatigue or emotional states, whereas AI anchors are not subject to such constraints (Liu et al., 2025). Highly customized functional features may further enable AI anchors to meet consumer needs in specific contexts (Niu et al., 2023). Given that responsiveness can enhance perceived usefulness and reduce perceived risk and psychological distance (Xue et al., 2020), AI anchors may facilitate purchase decisions by responding more quickly to consumer inquiries (Gao et al., 2023). Accordingly, we propose:

*H5*: Perceived responsiveness mediates the relationship between anchor type and trust, such that AI anchors elicit higher perceived responsiveness than human anchors.

### 2.4 Motivation orientations

Drawing on the uses-and-gratifications perspective, consumers’ participation in live-streaming commerce is driven by both hedonic and utilitarian gratifications (Mehmood et al., 2024). Hedonic motivation, also referred to as perceived enjoyment, is one of the primary drivers of media use. It conceptualizes enjoyment as reflecting the experiential and affective aspects of consumption, emphasizing consumers’ desire for relaxation, pleasure, and entertainment (Balabanis and Chatzopoulou, 2019). A substantial body of research has linked hedonic motivation to outcomes in live-streaming commerce. For example, hedonic motivation has been shown to explain consumers’ willingness to participate in live-stream shopping (Cai et al., 2018), while entertainment gratification enhances consumers’ loyalty to live-streaming channels (Hsu et al., 2020). Consumers may derive enjoyment from anchors’ language strategies and interactive engagement (Tseng and Wei, 2020), limited-time promotional strategies (Wongkitrungrueng and Assarut, 2020), and chat-based interaction (Wulf et al., 2020).

In contrast, utilitarian gratification refers to consumption that is goal-oriented, rational, and functionally driven (Dhar and Wertenbroch, 2000). Utilitarian-oriented consumers prioritize efficiency and instrumental value (Lee and Park, 2024), and their affective responses are more closely tied to cognitive evaluations and objective performance assessments (Liu et al., 2025). Accordingly, utilitarian gratification emphasizes practicality, problem-solving, and goal-directed outcomes (Guo et al., 2022; Tseng et al., 2017, 2018). As a result, utilitarian-oriented consumers tend to value predictable and rational benefits, whereas hedonic-oriented consumers are more responsive to affective evaluations such as enjoyment, fun, and emotional engagement (Kautish et al., 2023).

We propose that these two motivational orientations function as boundary conditions that shape how identity cues influence trust formation. Trust reflects consumers’ evaluative expectations toward anchors, and such expectations are closely tied to individual value orientations. Although both hedonic and utilitarian tendencies can enhance consumers’ trust in online shopping contexts (Mehmood et al., 2024), consumers with different motivational priorities may rely on distinct antecedents when forming trust judgments. Prior research has shown that consumers with different shopping values exhibit different emotional and evaluative patterns when interacting with anchors (Liu et al., 2025), suggesting that external stimuli are processed differently depending on the consumer’s active motivational state.

Specifically, we argue that hedonic and utilitarian motivations influence how consumers weight different identity cues during trust inference: hedonic motivation is more likely to amplify the influence of relational cues (perceived intimacy), whereas utilitarian motivation is more likely to amplify the influence of functional cues (perceived responsiveness). This implies that motivational orientations may not only exert direct effects on trust but also moderate the impact of identity cues on trust formation. Hedonic-oriented consumers, who emphasize experiential satisfaction, may exhibit stronger trust when higher levels of perceived intimacy are present. In contrast, utilitarian-oriented consumers, who prioritize product attributes and efficiency, may exhibit stronger trust when higher levels of perceived responsiveness are observed. Based on this reasoning, we propose the following hypotheses:

*H6*: Hedonic motivation positively moderates the effect of perceived intimacy on trust.

*H7*: Utilitarian motivation positively moderates the effect of perceived responsiveness on trust.

**Figure 1:**
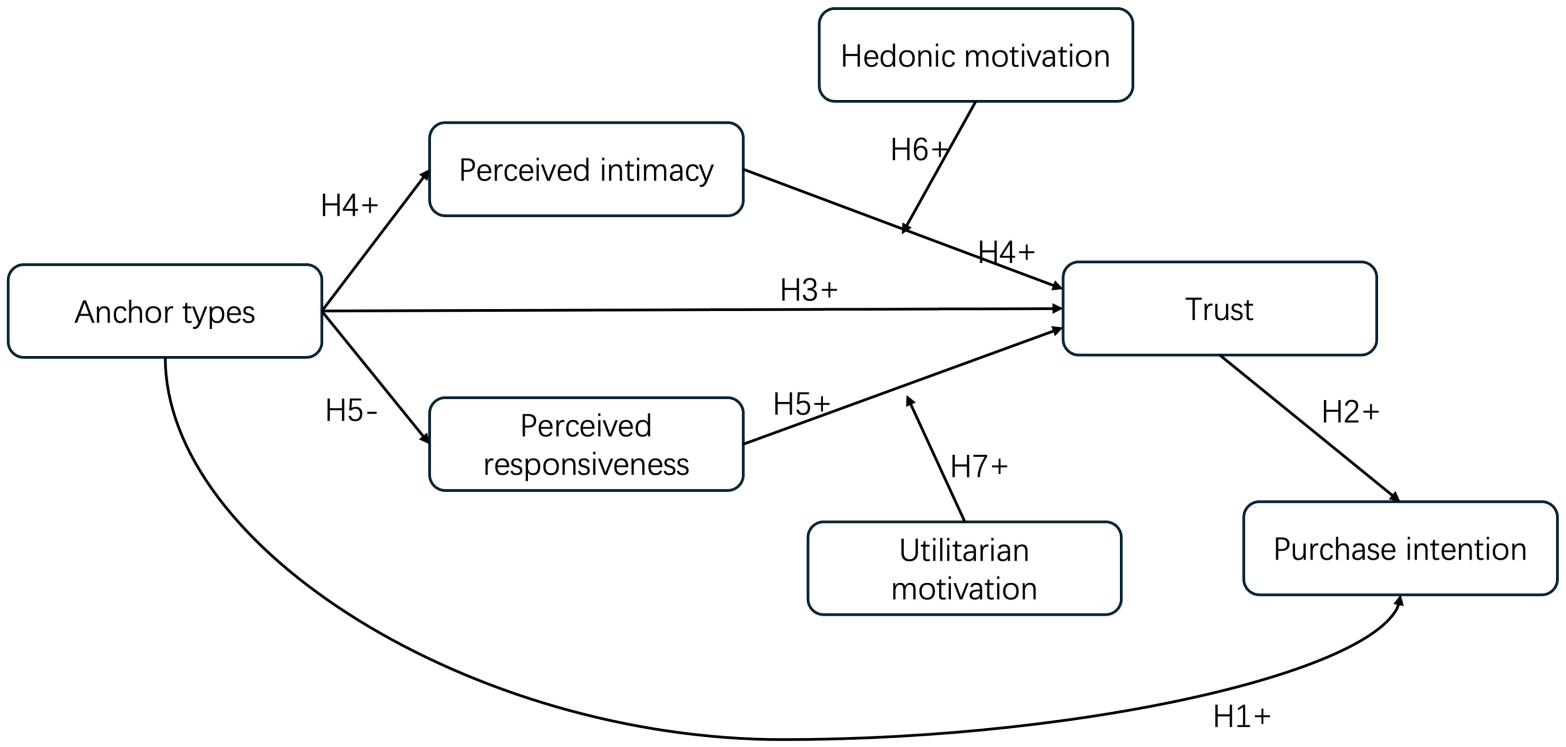
Theoretical model.

The theoretical model of this study is shown in Figure 1.

## 3 Methods

### 3.1 Procedure and participants

This study employed a between-subjects experimental design to examine the effects of anchor type (AI anchor vs. human anchor) on consumer trust and purchase intention in the context of live-streaming commerce. Participants were recruited through a professional online survey platform in China. Eligibility criteria required participants to (a) be at least 18 years old and (b) have prior experience watching live-streaming commerce. After accessing the survey, participants first read an informed consent form and indicated their voluntary agreement to participate. They then completed screening questions confirming that they had watched live-streaming commerce within the past 6 months and had prior exposure to either human or AI anchors.

Before exposure to the experimental stimulus, participants completed pre-stimulus measures assessing hedonic motivation, utilitarian motivation, and behavioral background variables (including live-streaming watching frequency, purchase frequency, and familiarity with AI virtual anchors). These variables were treated as relatively stable individual characteristics and were measured prior to stimulus exposure to avoid post-stimulus contamination. Participants were then randomly assigned to one of two experimental conditions. In each condition, participants watched a short live-streaming sales video hosted either by an AI anchor or a human anchor. Participants were required to view the video in full before proceeding.

After viewing the stimulus, participants completed an attention check, followed by manipulation check items assessing perceived anchor identity. They then responded to post-stimulus measures of perceived intimacy, perceived responsiveness, trust, and purchase intention. Finally, participants provided demographic information and were debriefed. Participants were excluded from analysis if they (a) failed the attention check or (b) provided incomplete responses. After applying these criteria, the final sample consisted of *N* = 439 participants.

This study was reviewed and approved by the Ethics Committee of the School of Journalism and Communication at Chongqing University (Approval No. CQUSJC2026007). All participants provided informed consent prior to participation. Table 1 presents the demographic characteristics and live-streaming behavioral profiles of the research sample.

**Table 1:** Descriptive statistics of the study sample.

Sample characteristics	Classification	n	%
Gender	Male	217	49.43
	Female	222	50.57
Anchor type	AI anchor	220	50.11
	Human anchor	219	49.89
Age	18–25	33	7.52
	26–30	221	50.34
	31–40	155	35.31
	41–50	30	6.83
Live-streaming watching frequency	1 (Never)	22	5.01
	2 (Rarely)	21	4.78
	3 (Occasionally)	45	10.25
	4 (Sometimes)	36	8.20
	5 (Often)	83	18.91
	6 (Very often)	89	20.27
	7 (Almost daily)	143	32.57
Live-streaming purchase frequency	1 (Never)	15	3.42
	2 (Rarely)	19	4.33
	3 (Occasionally)	41	9.34
	4 (Sometimes)	42	9.57
	5 (Often)	92	20.96
	6 (Very often)	98	22.32
	7 (Almost daily)	132	30.07

### 3.2 Stimulus materials

To manipulate anchor type, two live-streaming sales video clips were selected from existing live-streaming commerce sessions rather than being self-produced. One clip featured a human anchor, and the other featured an AI virtual anchor. Both clips promoted products within the same product category and reflected typical live-streaming sales contexts. The two clips were selected to ensure close comparability in terms of sales script, product information, presentation structure, and overall promotional framing. To isolate the effect of anchor type, the two video clips were selected to be broadly comparable in terms of video length, information content, speaking pace, and overall presentation flow. Brand cues, pricing information, and visual layout were highly similar, though not identical, across conditions.

Specifically, the two video clips were sourced from live-streaming commerce sessions featuring Luo Yonghao, a well-known Chinese internet celebrity. The human-anchor stimulus featured Luo Yonghao himself presenting a promotional offer for carbonated beverages, a choice of Coca-Cola, Fanta, or Sprite (approximately 2 min 40 s). The AI-anchor stimulus featured a digital avatar modelled on Luo Yonghao, presenting a promotional offer for carbonated beverages (approximately 3 min 13 s). Both clips promoted the same product category (carbonated beverages from the same brand portfolio) within a promotional framing, though the specific price points and bundle sizes differed across conditions. This difference reflects the ecological reality of live-streaming commerce, where AI-hosted sessions frequently employ aggressive promotional pricing to attract first-time viewers, while human-hosted sessions offer standard promotional bundles. Because both offers represented clear value propositions within the same low-involvement product category, the pricing variation is unlikely to have differentially activated hedonic or utilitarian processing beyond what would naturally occur in authentic live-streaming contexts. The AI avatar was developed by learning from and replicating the real anchor’s expressive patterns; its tone of voice, clothing, and facial features were designed to closely match those of the human anchor, though perfect replication was not achievable given current AI technology. Importantly, this residual discrepancy is inherent to the construct of anchor type itself. The experimental manipulation of anchor type is not intended to isolate a single perceptual dimension (e.g., voice naturalness alone); rather, it captures the holistic bundle of cues including subtle differences in facial expressiveness, vocal naturalness, and physical fluidity, that consumers encounter when exposed to an AI versus human anchor in real-world live-streaming contexts. As such, any differences in perceived attractiveness, voice quality, or gestural naturalness between conditions are conceptually embedded within the anchor-type variable rather than representing extraneous confounds, consistent with the view that users respond to media agents as integrated social entities rather than processing individual perceptual features in isolation (Reeves and Nass, 1996). The slight difference in video duration (33 s) was deemed negligible relative to the overall length and did not affect the quantity or structure of product information conveyed.

It should be noted that because the stimuli were drawn from authentic live-streaming sessions rather than self-produced, the two videos were comparable but not identical. Although the clips were selected to match in script structure, information content, and overall presentation flow, uncontrolled differences in delivery style, production quality, or anchor-specific features may exist. This design choice prioritized ecological validity at the cost of some experimental control. The implications of this trade-off are addressed in the limitations section.

### 3.3 Measures

Unless otherwise specified, all constructs were measured using 7-point Likert-type scales (1 = strongly disagree, 7 = strongly agree). Item scores were averaged to form composite indices for subsequent analyses. All continuous predictors were mean-centered prior to hypothesis testing to facilitate interpretation of interaction effects and reduce multicollinearity.

Hedonic motivation reflects enjoyment-oriented and experiential shopping goals in the context of live-streaming commerce. This construct was measured using four items adapted from Liu et al. (2025). Participants indicated the extent to which each statement described their usual experience when watching live-streaming commerce. Sample items include “Watching live-streaming commerce is exciting to me” and “I usually feel happy when watching live-streaming commerce.”

Utilitarian motivation captures efficiency-oriented and goal-directed considerations underlying consumers’ engagement with live-streaming commerce. This variable was measured using four items adapted from Liu et al. (2025). Sample items include “Watching live-streaming commerce helps me identify products that meet my needs” and “Buying products through live-streaming commerce is convenient.”

Anchor type served as the experimental independent variable and was manipulated at two levels: a human anchor and an AI virtual anchor. For analytical purposes, anchor type was dummy-coded, with the AI anchor coded as 0 and the human anchor coded as 1.

Perceived intimacy represents a relational cue reflecting emotional closeness and parasocial connection between the participant and the anchor. This construct was measured using five items adapted from Lee and Choi (2017). Sample items include “I feel that I am close to the live streamer” and “I feel emotionally close to the live streamer.”

Perceived responsiveness reflects a functional cue capturing the anchor’s perceived ability to respond efficiently and adequately to audience needs during the live-streaming session. This variable was measured using three items adapted from Yuan et al. (2022). Items include “I obtain information from the live streamer in a timely manner,” “The live streamer responds very quickly to my feedback,” and “The live streamer processes my input efficiently.” Because participants viewed pre-recorded video clips rather than participating in live interactive sessions, perceived responsiveness in this study reflects participants’ inferences about the anchor’s capacity for responsive interaction, based on observable cues in the video (e.g., pace, apparent attentiveness, information delivery style), rather than direct interactive experience.

Trust was conceptualized as a proximal psychological outcome reflecting participants’ expectations regarding the credibility, reliability, and trustworthiness of the anchor and the product-related information provided. Trust was measured using three items adapted from Meng et al. (2021) and Chua et al. (2008). Sample items include “I believe that the live streamer is credible” and “I trust the product information provided by the live streamer.”

Purchase intention was conceptualized as the downstream behavioral outcome following trust formation. This construct was measured using four items adapted from Chen et al. (2022), capturing participants’ interest, willingness, and likelihood of purchasing the product presented in the live-streaming session. Sample items include “After viewing this live streaming, I became interested in making a purchase” and “After viewing this live streaming, I am likely to buy the product.”

Several control variables were included to account for alternative explanations, including gender, live-streaming watching frequency, and live-streaming purchase frequency. All control variables were included as covariates in the analyses rather than as focal predictors.

### 3.4 Analytic strategy

Data analyses were conducted using Python 3.12 with the SciPy, semopy, and NumPy libraries, replicating the analytic logic of the PROCESS macro (Model 14; Hayes, 2022). Composite indices were constructed by averaging corresponding items. All continuous predictors were mean-centered before analysis to facilitate interpretation of interaction effects and reduce multicollinearity.

The analytic procedure proceeded in five stages. First, confirmatory factor analysis (CFA) was conducted to evaluate the measurement model. Second, reliability and convergent validity were assessed using Cronbach’s alpha, composite reliability (CR), and average variance extracted (AVE). Third, discriminant validity was evaluated using the Fornell–Larcker criterion and heterotrait-monotrait (HTMT) ratios. Fourth, group equivalence between experimental conditions was tested using chi-square tests and independent-samples *t*-tests. Fifth, hypothesis testing was conducted using OLS regression with heteroscedasticity-consistent (HC3) standard errors, following the moderated mediation framework. Common method bias was assessed using Harman’s single-factor test.

## 4 Results

### 4.1 Measurement model

#### 4.1.1 Confirmatory factor analysis

A six-factor CFA was estimated using maximum likelihood estimation. The hypothesized model included hedonic motivation (4 items), utilitarian motivation (4 items), perceived intimacy (5 items), perceived responsiveness (3 items), trust (3 items), and purchase intention (4 items). The model demonstrated acceptable fit: χ^2^(215) = 728.15, *p* < 0.001; CFI = 0.946; TLI = 0.937; RMSEA = 0.074; GFI = 0.926. Although the chi-square test was significant, this is expected given the large sample size (*N* = 439; Hu and Bentler, 1999). The CFI and TLI values exceeded the recommended 0.90 threshold, and RMSEA fell below 0.08, indicating adequate model fit.

#### 4.1.2 Reliability and convergent validity

Table 2 presents the standardized factor loadings, reliability coefficients, composite reliability, and average variance extracted for all constructs. All standardized factor loadings ranged from 0.798 to 0.930, exceeding the 0.70 threshold. Internal consistency reliability was evaluated using Cronbach’s alpha; all values ranged from 0.909 to 0.965, well above the 0.70 threshold. Composite reliability (CR) values ranged from 0.936 to 0.972, all exceeding 0.70. Average variance extracted (AVE) values ranged from 0.785 to 0.904, all exceeding the 0.50 threshold recommended by Hair et al. (2019). These results indicate satisfactory convergent validity across all constructs.

**Table 2:** Standardized factor loadings, reliability, composite reliability, and AVE.

Construct	Item	λ	α	CR	AVE
Hedonic motivation			0.909	0.936	0.785
	HM-1	0.862			
	HM-2	0.798			
	HM-3	0.876			
	HM-4	0.842			
Utilitarian motivation			0.915	0.940	0.798
	UM-1	0.861			
	UM-2	0.854			
	UM-3	0.851			
	UM-4	0.851			
Perceived intimacy			0.965	0.972	0.876
	PI-1	0.921			
	PI-2	0.896			
	PI-3	0.925			
	PI-4	0.927			
	PI-5	0.928			
Perceived responsiveness			0.947	0.966	0.904
	PR-1	0.930			
	PR-2	0.930			
	PR-3	0.917			
Trust			0.914	0.946	0.854
	TR-1	0.879			
	TR-2	0.875			
	TR-3	0.897			
Purchase intention			0.924	0.946	0.814
	PI-1	0.864			
	PI-2	0.859			
	PI-3	0.872			
	PI-4	0.873			

λ, standardized factor loading from CFA; α, Cronbach’s alpha; CR, composite reliability; AVE, average variance extracted. CFA model fit: χ^2^(215) = 728.15, *p* < 0.001; CFI = 0.946; TLI = 0.937; RMSEA = 0.074; GFI = 0.926.

#### 4.1.3 Discriminant validity

Discriminant validity was assessed using two complementary criteria. First, the Fornell–Larcker criterion was applied: for every construct pair, the square root of each construct’s AVE (shown on the diagonal of Table 3) exceeded its correlation with every other construct, confirming adequate discriminant validity. Second, heterotrait-monotrait (HTMT) ratios were computed; all values ranged from 0.046 to 0.690, well below the conservative 0.85 threshold recommended by Henseler et al. (2015). Table 3 presents the inter-construct correlation matrix, and Table 4 reports the HTMT values.

**Table 3:** Correlation Matrix with √AVE on the Diagonal.

Variable	HM	UM	PI	PR	TR	PuI
HM	**0.886**					
UM	0.117	**0.893**				
PI	0.235	0.109	**0.936**			
PR	0.115	0.083	−0.301	**0.951**		
TR	0.629	0.116	0.415	0.013	**0.924**	
PuI	0.347	0.173	0.443	0.067	0.424	**0.902**

Diagonal values (bold) represent the square root of AVE. Off-diagonal values are Pearson correlations between construct composites. HM, Hedonic Motivation; UM, Utilitarian Motivation; PI, Perceived Intimacy; PR, Perceived Responsiveness; TR, Trust; PuI, Purchase Intention.

**Table 4:** Heterotrait-Monotrait (HTMT) ratios.

Pair	HTMT	Pair	HTMT	Pair	HTMT
HM–UM	0.129	UM–PR	0.094	PI–PuI	0.469
HM–PI	0.250	UM–TR	0.127	PR–TR	0.046
HM–PR	0.132	UM–PuI	0.188	PR–PuI	0.072
HM–TR	0.690	PI–PR	0.315	TR–PuI	0.461
HM–PuI	0.379	PI–TR	0.441		
UM–PI	0.116				

All HTMT values < 0.85 conservative threshold, confirming discriminant validity.

### 4.2 Common method bias

Because all self-report measures were collected in a single survey session, common method bias (CMB) was assessed using Harman’s single-factor test (Podsakoff et al., 2003). An unrotated principal component analysis including all 23 measurement items was conducted. The first factor accounted for 33.11% of total variance, well below the 50% threshold. This suggests that CMB does not pose a serious threat to the validity of the findings.

### 4.3 Manipulation check

Two manipulation check measures were used to assess the effectiveness of the anchor-type manipulation. First, participants identified the anchor’s identity on a 5-point scale ranging from “definitely an AI virtual anchor” to “definitely a human anchor.” Participants selecting “not sure” were excluded from subsequent analyses. Second, participants evaluated the perceived humanness and artificiality of the anchor.

Independent-samples t-tests confirmed that the manipulation was successful. Participants in the human anchor condition rated the anchor as significantly more humanlike (M = 6.00, SD = 0.80) than those in the AI anchor condition (M = 4.40, SD = 1.53), t(437) = 13.72, *p* < 0.001, d = 1.31. Conversely, participants in the AI anchor condition rated the anchor as significantly more automated (M = 4.21, SD = 1.62) than those in the human anchor condition (M = 1.71, SD = 0.72), t(437) = −20.92, *p* < 0.001, d = −2.00. These large effect sizes confirm that participants clearly differentiated between the two anchor types. Notably, the AI anchor scored above the scale midpoint on perceived humanness (M = 4.40 on a 7-point scale), suggesting that participants perceived it as possessing some social-agent qualities consistent with the Computers Are Social Actors framework (Nass and Moon, 2000), even while recognizing it as AI.

### 4.4 Randomization check

To verify that random assignment produced comparable experimental groups, chi-square tests were conducted on categorical demographic variables and independent-samples *t*-tests on pre-stimulus continuous measures. As shown in Table 5, no significant differences were found between the AI anchor (*n* = 220) and human anchor (*n* = 219) conditions on gender (χ^2^(1) = 0.002, *p* = 0.962), age (χ^2^(3) = 3.636, *p* = 0.304), education (χ^2^(4) = 4.835, *p* = 0.305), income (χ^2^(5) = 5.670, *p* = 0.340), watching frequency (χ^2^(6) = 3.753, *p* = 0.710), or purchase frequency (χ^2^(6) = 5.301, *p* = 0.506).

**Table 5:** Group equivalence tests.

Variable	AI anchor	Human anchor	Test	p
Gender (% female)	50.9%	50.2%	χ^2^(1) = 0.002	0.962
Age group	—	—	χ^2^(3) = 3.636	0.304
Education	—	—	χ^2^(4) = 4.835	0.305
Income	—	—	χ^2^(5) = 5.670	0.340
Watching frequency	—	—	χ^2^(6) = 3.753	0.710
Purchase frequency	—	—	χ^2^(6) = 5.301	0.506
Hedonic motivation	4.71 (1.24)	4.96 (1.20)	t(437) = −2.16	0.031*
Utilitarian motivation	4.84 (1.26)	4.79 (0.95)	t(437) = 0.49	0.624
AI familiarity	4.95 (1.79)	5.26 (1.75)	t(437) = −1.84	0.067

*p < 0.05. Continuous variables reported as M (SD). Categorical variables tested via chi-square; continuous variables via independent-samples *t*-tests.

Independent-samples t-tests further indicated no significant differences in utilitarian motivation (AI: M = 4.84, SD = 1.26; Human: M = 4.79, SD = 0.95; t(437) = 0.49, *p* = 0.624) or AI anchor familiarity (AI: M = 4.95, SD = 1.79; Human: M = 5.26, SD = 1.75; t(437) = −1.84, *p* = 0.067). However, hedonic motivation was marginally higher in the human anchor condition (AI: M = 4.71, SD = 1.24; Human: M = 4.96, SD = 1.20; t(437) = −2.16, *p* = 0.031, d = 0.21). To address this, hedonic motivation was included as a moderator in all mediation and moderation analyses, and the substantive pattern of results remained unchanged when hedonic motivation was additionally controlled in the direct-effect models.

### 4.5 Descriptive statistics

Table 6 presents the descriptive statistics for all focal variables, both in original and mean-centered form. All variables showed sufficient variability (SD > 1.0) to support subsequent hypothesis testing. Skewness values ranged from −0.26 to 0.20, and kurtosis values ranged from −1.10 to −0.43, all falling within acceptable ranges for parametric analyses.

**Table 6:** Descriptive statistics for focal variables.

Variable	M	SD	Min	Max	Skew	Kurt
Hedonic motivation	4.84	1.22	1.25	7.00	−0.26	−0.43
Utilitarian motivation	4.81	1.11	2.25	7.00	0.20	−0.64
Perceived intimacy	4.37	1.57	1.00	7.00	−0.14	−0.92
Perceived responsiveness	3.93	1.73	1.00	7.00	0.05	−1.10
Trust	4.50	1.43	1.00	7.00	−0.18	−0.53
Purchase intention	4.44	1.33	1.00	7.00	−0.09	−0.49

### 4.6 Direct effects

H1 predicted that human anchors would generate higher purchase intention than AI anchors at an overall level. Purchase intention was regressed on anchor type, controlling for gender, watching frequency, and purchase frequency. As reported in Table 7, anchor type exerted a significant positive total effect on purchase intention (b = 0.54, SE = 0.13, t = 4.34, *p* < 0.001, 95% CI [0.30, 0.79]), indicating that consumers exposed to human anchors reported significantly higher purchase intention than those exposed to AI anchors. The mean difference between conditions was 0.54, corresponding to a moderate effect size (Cohen’s d = 0.41). Thus, H1 was supported.

**Table 7:** Regression results for direct effects.

Predictor	Outcome	b	SE(HC3)	t	*p*	95% CI	R^2^
Anchor type	Purchase intention	0.54	0.13	4.34	< 0.001	[0.30, 0.79]	0.050
Anchor type	Trust	0.78	0.13	5.90	< 0.001	[0.52, 1.04]	0.083
Trust	Purchase intention	0.37	0.05	7.01	< 0.001	[0.26, 0.47]	0.192

Anchor type coded 0 = AI anchor, 1 = human anchor. All models control for gender, watching frequency, and purchase frequency.

H2 proposed that trust in the anchor would positively influence consumers’ purchase intention. When purchase intention was regressed on trust (mean-centered) and anchor type with controls, trust showed a significant positive effect (b = 0.37, SE = 0.05, t = 7.01, *p* < 0.001, 95% CI [0.26, 0.47]). The model explained 19.2% of variance in purchase intention. Therefore, H2 was supported.

H3 further predicted that human anchors would elicit higher trust than AI anchors. Results confirmed this prediction (b = 0.78, SE = 0.13, t = 5.90, *p* < 0.001, 95% CI [0.52, 1.04]). The mean difference was 0.77 (95% CI [0.51, 1.03]), with a moderate effect size (Cohen’s d = 0.56). The model accounted for 8.3% of variance in trust. H3 was supported. Table 8 summarizes the group differences and effect sizes for all focal variables by anchor type.

**Table 8:** Group differences and effect sizes by anchor type.

Variable	AI (*n* = 220)	Human (*n* = 219)	Diff [95% CI]	Cohen’s d
Perceived intimacy	3.35 (1.24)	5.40 (1.14)	2.06 [1.83, 2.28]	1.72
Perceived responsiveness	5.12 (1.31)	2.74 (1.21)	−2.38 [−2.62, −2.15]	−1.89
Trust	4.11 (1.34)	4.88 (1.42)	0.77 [0.51, 1.03]	0.56
Purchase intention	4.17 (1.24)	4.71 (1.36)	0.54 [0.30, 0.78]	0.41

Values are M (SD). Diff = Human − AI. Effect size benchmarks: 0.20 = small, 0.50 = medium, 0.80 = large.

### 4.7 Mediating effects

H4 proposed that perceived intimacy mediates the relationship between anchor type and trust. Results from the a-path regression indicated that anchor type significantly predicted perceived intimacy (b = 2.07, SE = 0.11, t = 18.43, *p* < 0.001, 95% CI [1.85, 2.29], R^2^ = 0.450), confirming that human anchors elicited substantially higher perceived intimacy than AI anchors (mean difference = 2.06, 95% CI [1.83, 2.28], d = 1.72; see Table 9, Panel A).In the trust equation, which included both mediators, both moderators, their interactions, and all covariates (see Table 9), perceived intimacy was positively associated with trust at the mean level of hedonic motivation (b = 0.20, SE = 0.05, t = 3.90, *p* < 0.001, 95% CI [0.10, 0.30]). However, as hypothesized, this association was conditional on consumers’ hedonic motivation.

**Table 9:** Mediation and conditional indirect effects of anchor type on trust.

Pathway	b/effect	SE	t	*p*	95% CI
Panel A: path coefficients (a paths)					
Anchor type → perceived intimacy	2.07	0.11	18.43	< 0.001	[1.85, 2.29]
Anchor type → perceived responsiveness	−2.37	0.12	−19.66	< 0.001	[−2.61, −2.14]
Panel B: conditional indirect effects					
Via PI (low hedonic)	0.24				
Via PI (mean hedonic)	0.44				
Via PI (high hedonic)	0.63				
Via PR (low utilitarian)	0.16				
Via PR (mean utilitarian)	−0.25				
Via PR (high utilitarian)	−0.67				
Index of moderated mediation					
Via PI (by HM)	0.16				
Via PR (by UM)	−0.37				

Low = M − 1SD; Mean = M; High = M + 1SD. Anchor type: 0 = AI, 1 = human.

The conditional indirect effect of anchor type on trust via perceived intimacy was not significant at low hedonic motivation (indirect effect = 0.24), became significant at the mean (indirect effect = 0.44), and was strongest at high hedonic motivation (indirect effect = 0.63; see Table 8, Panel B). These results indicate that perceived intimacy mediated the effect of anchor type on trust only when consumers’ hedonic motivation was moderate to high. Thus, H4 was conditionally supported.

H5 examined perceived responsiveness as an alternative mediator, proposing that AI anchors would elicit higher perceived responsiveness than human anchors, which would subsequently influence trust. The a-path regression confirmed that anchor type significantly predicted perceived responsiveness (b = −2.37, SE = 0.12, t = −19.66, *p* < 0.001, 95% CI [−2.61, −2.14], R^2^ = 0.477), with AI anchors perceived as substantially more responsive (mean difference = −2.38, d = −1.89; Table 9, Panel A).In the full trust model, the association between perceived responsiveness and trust was significant at the mean level of utilitarian motivation (b = 0.11, SE = 0.05, t = 2.24, *p* = 0.026, 95% CI [0.01, 0.20]). However, the conditional indirect effects revealed a more complex pattern. The indirect effect was positive (favoring human anchors) at low utilitarian motivation (indirect effect = 0.16), became negative at the mean (indirect effect = −0.25), and was strongly negative (favoring AI anchors) at high utilitarian motivation (indirect effect = −0.67; Table 9, Panel B). This reversal indicates that perceived responsiveness does not function as a universal mediator; rather, it operates as a fully conditional mechanism that favors AI anchors only among highly utilitarian-motivated consumers. Therefore, H5 was not supported as a general mechanism; instead, the results point to a fully conditional mediation process in which perceived responsiveness serves as a trust-building pathway for AI anchors only among consumers with high utilitarian motivation. This asymmetry between the two mediating pathways is a central finding of the study.

### 4.8 Moderating effects

Table 10 reports the full moderated mediation model predicting trust. The overall model explained 53.2% of the variance in trust (R^2^ = 0.532, *F*(10, 428) = 48.73, *p* < 0.001).

**Table 10:** Moderated mediation model predicting trust (full model).

Predictor	b	SE(HC3)	t	*p*	95% CI
Constant	4.24	0.21	19.76	< 0.001	[3.82, 4.66]
Anchor type	0.53	0.23	2.31	0.022	[0.08, 0.98]
Perceived intimacy (centered)	0.20	0.05	3.90	< 0.001	[0.10, 0.30]
Hedonic motivation (centered)	0.54	0.05	10.17	< 0.001	[0.43, 0.64]
PI × HM	0.07	0.03	2.58	0.010	[0.02, 0.13]
Perceived responsiveness (centered)	0.11	0.05	2.24	0.026	[0.01, 0.20]
Utilitarian motivation (centered)	−0.02	0.05	−0.39	0.699	[−0.13, 0.08]
PR × UM	0.16	0.03	5.00	< 0.001	[0.09, 0.22]
Gender	−0.02	0.10	−0.17	0.867	[−0.22, 0.18]
Watching frequency	−0.01	0.03	−0.20	0.842	[−0.07, 0.06]
Purchase frequency	−0.00	0.04	−0.11	0.912	[−0.07, 0.07]

R^2^ = 0.532, F(10, 428) = 48.73, *p* < 0.001. All continuous predictors mean-centered.

H6 predicted that hedonic motivation would positively moderate the effect of perceived intimacy on trust. The interaction between perceived intimacy and hedonic motivation was statistically significant (b = 0.07, SE = 0.03, t = 2.58, *p* = 0.010, 95% CI [0.02, 0.13]), indicating that the effect of perceived intimacy on trust varied as a function of hedonic motivation. As shown in Table 10, perceived intimacy was not significantly associated with trust at low hedonic motivation (b = 0.11, *p* = 0.075), showed a significant positive association at the mean (b = 0.20, *p* < 0.001), and exhibited the strongest positive association at high hedonic motivation (b = 0.29, *p* < 0.001). The index of moderated mediation was 0.16, confirming that the indirect effect of anchor type on trust via perceived intimacy increased as hedonic motivation rose. H6 was supported.

H7 proposed that utilitarian motivation would positively moderate the effect of perceived responsiveness on trust. The interaction between perceived responsiveness and utilitarian motivation was highly significant (b = 0.16, SE = 0.03, t = 5.00, *p* < 0.001, 95% CI [0.09, 0.22]). As shown in Table 11, perceived responsiveness was negatively (non-significantly) associated with trust at low utilitarian motivation (b = −0.07, *p* = 0.251), significantly positive at the mean (b = 0.11, *p* = 0.026), and strongly positive at high utilitarian motivation (b = 0.28, *p* < 0.001). Given the coding scheme (0 = AI, 1 = human), the negative index of moderated mediation (−0.37) indicates that the conditional indirect pathway increasingly favored AI anchors at higher levels of utilitarian motivation. H7 was supported.

**Table 11:** Conditional effects of perceived intimacy and responsiveness on trust.

Moderator level	Effect of PI on trust	SE	*p*	95% CI	Effect of PR on trust	SE	*p*	95% CI
Low (M − 1SD)	0.11	0.06	0.075	[−0.01, 0.23]	−0.07	0.06	0.251	[−0.18, 0.05]
Mean (M)	0.20	0.05	< 0.001	[0.10, 0.30]	0.11	0.05	0.026	[0.01, 0.20]
High (M + 1SD)	0.29	0.06	< 0.001	[0.17, 0.41]	0.28	0.06	< 0.001	[0.16, 0.39]

The moderator for PI is hedonic motivation; the moderator for PR is utilitarian motivation.

Taken together, the moderation results reveal an asymmetric pattern. The relational pathway (perceived intimacy, moderated by hedonic motivation) operates as a relatively broad mediator, reaching significance at average and high levels of hedonic motivation. The functional pathway (perceived responsiveness, moderated by utilitarian motivation) operates as a narrower, conditional mediator that reaches significance only at high utilitarian motivation. This asymmetry has implications for the overall pattern of findings, which are elaborated in the Discussion.

### 4.9 Robustness and sensitivity analyses

A series of robustness and sensitivity analyses were conducted to evaluate the stability of the findings. First, all regression and moderated mediation analyses employed heteroscedasticity-consistent (HC3) standard errors throughout, providing robustness against potential violations of homoscedasticity. The direction, magnitude, and statistical significance of all focal effects remained consistent under this conservative estimation strategy.

Second, sensitivity analyses were performed by re-estimating the total-effect models using z-standardized versions of the dependent variables. Anchor type continued to exert significant positive effects on standardized purchase intention (*β* = 0.41, t = 4.34, *p* < 0.001) and standardized trust (*β* = 0.55, *t* = 5.90, *p* < 0.001), indicating that the substantive conclusions were not sensitive to variable scaling.

Third, multicollinearity diagnostics revealed no concerns: all variance inflation factor (VIF) values in the full moderated mediation model ranged from 1.09 to 3.35, well below the conventional threshold of 5. The highest VIF (3.35 for anchor type) reflects its expected collinearity with the two mediators, which are strongly differentiated by condition, but does not reach problematic levels.

Fourth, the Cohen’s f^2^ effect sizes for anchor type in the direct-effect models were 0.044 (on purchase intention) and 0.082 (on trust), corresponding to small and small-to-medium effects, respectively (Cohen, 1988). While these effect sizes are modest, they are consistent with effect sizes typically observed in experimental studies of consumer behavior in mediated contexts. The larger effect sizes observed for the mediation pathways (d = 1.72 for intimacy, d = −1.89 for responsiveness) indicate that anchor type exerts its influence primarily through these identity-based cue mechanisms rather than directly. Table 12 is summary of hypothesis tests.

**Table 12:** Summary of Hypothesis Tests.

Hypothesis	Path	Result
H1	Human anchors → higher purchase intention	Supported
H2	Trust → purchase intention (+)	Supported
H3	Human anchors → higher trust	Supported
H4	Anchor type → intimacy → trust (mediation)	Conditionally supported
H5	Anchor type → responsiveness → trust (mediation)	Not supported as general mechanism; conditional mediation favoring AI anchors only at high utilitarian motivation
H6	HM moderates PI → Trust	Supported
H7	UM moderates PR → Trust	Supported

## 5 Discussion

### 5.1 Summary of findings

The present study examined how anchor type (AI versus human) shapes consumer trust and purchase intention in live-streaming commerce. Rather than treating this as a question of marketing efficiency, the study approached it as a question of media psychology and human–machine communication: how do consumers, as media users, psychologically process AI versus human communicators in an interactive media environment, and through which identity-based cue pathways does each type of communicator build trust?

At the overall level, human anchors generated higher trust and purchase intention than AI anchors. This finding is consistent with the broader pattern of algorithm aversion documented in the psychology of AI literature, whereby individuals tend to exhibit reluctance toward algorithmic agents compared to human agents across a wide range of evaluative and behavioral outcomes (Williams and Lim, 2024). It is also consistent with prior live-streaming research showing that human anchors hold persuasive advantages rooted in social presence and emotional expressiveness (Chen and Liao, 2022; Wongkitrungrueng and Assarut, 2020). Trust, in turn, was a significant predictor of purchase intention, confirming that in the uncertain environment of live-streaming commerce, consumers’ behavioral decisions are rooted in their credibility judgments of the anchor (Liu et al., 2025; Meng et al., 2021).

Crucially, the two identity-based cue pathways operated asymmetrically rather than in parallel. The relational pathway via perceived intimacy functioned as a relatively robust mediator: human anchors elicited substantially higher perceived intimacy, and this cue was positively associated with trust at average and high levels of hedonic motivation. By contrast, the functional pathway via perceived responsiveness did not operate as a general mediator. Although AI anchors held a pronounced advantage in perceived responsiveness, this advantage translated into trust only when consumers’ utilitarian motivation was high. At low utilitarian motivation, the indirect effect actually reversed direction, favoring human anchors. This asymmetry is the central finding of the study.

### 5.2 Theoretical implications

This study makes four contributions to the literature on human–AI interaction, media psychology, and live-streaming commerce.

First, the study reframes the AI replacement debate as a question about human–machine communication rather than commercial performance. The question of whether AI anchors can replace human anchors has generated considerable industry and academic attention (Liu et al., 2025), but the debate has been conducted largely in terms of observable outcomes, comparing sales figures, engagement metrics, or purchase intentions across anchor types (Gao et al., 2025; Gao et al., 2023). Such comparisons can document differences but cannot explain them. The present study shifts the analytical lens from “which anchor performs better” to “through which psychological pathways does each type of communicator build trust, and under what conditions.” This reframing is important because it recognizes that the replacement question is fundamentally a question about how people relate to different types of communicative agents in a mediated environment. The answer, as our findings suggest, is not that one type is superior, but that the two types activate different psychological mechanisms whose effectiveness depends on the consumer’s motivational context.

Second, the study offers a mechanism-based account of algorithm aversion in a live, interactive media context. Prior research has documented algorithm aversion as a general tendency, attributing it to perceived limitations in algorithms’ capacity to process qualitative information, to experience subjective states, and to exercise moral judgment (Williams and Lim, 2024). However, most of this research has examined algorithm aversion in static, non-interactive settings such as advice-taking, prediction, or hiring decisions. The present study extends this line of work to an interactive media context, live-streaming commerce, where the “algorithm” takes the form of a visible, speaking, socially engaging AI agent. Our findings suggest that algorithm aversion in this context is not uniform. Rather, it is mediated by specific identity cues: the AI anchor’s disadvantage in trust formation is traceable to lower perceived intimacy, which reflects consumers’ perception that the AI agent lacks emotional closeness and relational depth. This is consistent with the view that algorithm aversion stems partly from the perceived inability of AI agents to provide genuine subjective experience (Williams and Lim, 2024). At the same time, the AI anchor’s advantage in perceived responsiveness suggests that algorithm aversion is not absolute; in the functional domain, AI agents can outperform human agents in consumers’ eyes.

Third, and most importantly, the study reveals that the two trust pathways operate asymmetrically. The relational pathway via perceived intimacy functions as a relatively stable mediator, effective at moderate and high levels of hedonic motivation. The functional pathway via perceived responsiveness, by contrast, is a fully conditional mechanism that becomes effective only when utilitarian motivation is high. This asymmetry is the most novel finding of the study, and it has not been reported in prior research on AI anchors (Gao et al., 2025; Liu et al., 2025). It means that the two anchor types do not simply occupy different positions on a single continuum of effectiveness; rather, they operate through qualitatively different trust-building mechanisms with different activation thresholds. The intimacy cue is a broadly deployable trust resource, while the responsiveness cue is a specialist resource that requires motivational activation. This distinction has implications beyond live-streaming commerce: it suggests that in any context where human and AI communicators coexist, the psychological pathways through which each builds trust may differ not only in magnitude but in kind.

Fourth, the study establishes consumer motivation as a critical boundary condition for trust formation in human–machine communication. Prior research on trust in AI has recognized the importance of contextual factors (Omrani et al., 2022), and live-streaming research has examined hedonic and utilitarian motivations as predictors of behavior (Liu et al., 2025; Mehmood et al., 2024). However, these two bodies of work have not been integrated. The present study bridges them by showing that motivational orientations function not merely as predictors of trust, but as moderators that determine which cue pathway predominates in trust formation. This motivation-dependent cue-weighting process offers a principled answer to the replacement debate: AI anchors will not broadly replace human anchors, because the relational pathway that human anchors command is more broadly effective. But human anchors will not render AI anchors obsolete either, because the functional pathway that AI anchors command is highly effective for efficiency-oriented consumers. The relationship is conditional, not substitutional.

### 5.3 Explaining the overall human advantage

An apparent tension in the results is that human anchors held an overall advantage in trust and purchase intention despite AI anchors’ strong advantage in perceived responsiveness. The asymmetry between the two mediating pathways explains this pattern. The intimacy pathway has a higher and more stable “conversion rate” from cue to trust: human anchors’ large intimacy advantage combines with a consistently significant path to trust across moderate and high hedonic motivation. By contrast, AI anchors’ even larger responsiveness advantage pairs with a path to trust that is weak or non-significant except at high utilitarian motivation. In effect, the intimacy cue is a broadly deployable trust resource, while the responsiveness cue is a specialist resource that requires motivational activation. Because the average consumer in a live-streaming context is likely to carry at least moderate hedonic motivation, the intimacy pathway fires more reliably than the responsiveness pathway, producing the observed overall human advantage.

This finding has implications for how we understand algorithm aversion. Rather than being a blanket tendency, algorithm aversion in interactive media contexts appears to be a consequence of a structural mismatch: the cue pathway that AI agents command (functional responsiveness) has a narrower activation threshold than the pathway that human agents command (relational intimacy). When the motivational context aligns with the functional pathway, algorithm aversion diminishes or reverses. This suggests that algorithm aversion is, at least in part, a context-dependent phenomenon shaped by the interaction between agent identity cues and user motivational states.

### 5.4 Practical implications

The findings provide actionable strategies for brands and platforms in live-streaming commerce. First, anchor selection should be strategically aligned with consumer goals rather than defaulting to either anchor type. For utilitarian, information-intensive products (e.g., electronics, household appliances), AI anchors may be highly effective because their advantage in perceived responsiveness aligns with efficiency-oriented goals. For hedonic or lifestyle products (e.g., fashion, cosmetics), human anchors remain the preferred choice because their ability to generate perceived intimacy is essential for emotional engagement and trust formation.

Second, AI deployment should be treated as an identity design challenge, not merely a technological upgrade. Simply making AI anchors look more humanlike is insufficient if the underlying cue structure does not match the target audience’s motivational profile. If the target audience values efficiency, AI anchor design should emphasize speed, accuracy, and clear information delivery (functional cues). If the goal is social interaction and community building, the design must prioritize warmth, empathy, and conversational fluency (relational cues), areas where AI anchors currently face inherent limitations.

Third, platforms can leverage user data to match anchors with viewing contexts. Since the value of perceived responsiveness is conditional on utilitarian motivation, platforms can use browsing behavior and purchase history to infer motivational orientation. Users exhibiting utilitarian browsing patterns (e.g., direct product searches, comparison behavior) could be directed to AI-hosted streams with standardized, rapid information delivery. Users exhibiting hedonic browsing patterns (e.g., extended browsing, entertainment-seeking) could be directed to human-hosted streams to maximize relational value.

### 5.5 Limitations and future directions

Despite its contributions, this study has several limitations that should be considered when interpreting the results.

First, the study used a controlled experiment with pre-recorded video clips rather than live, interactive sessions. While this design ensured internal validity, it limited real-time interactivity. In particular, perceived responsiveness was assessed based on participants’ inferences from observable video cues (e.g., pace, apparent attentiveness, information delivery style) rather than from actual real-time interaction. In authentic live-streaming contexts, responsiveness is experienced directly through real-time question-answering and personalized feedback. The present design may therefore underestimate the true potential of AI anchors in interactive settings, where their algorithmic speed and consistency advantages could more fully manifest. Future research should examine these effects in live, fully interactive field environments to enhance ecological validity.

Second, the study used one pre-recorded video per experimental condition. Although the two stimuli were carefully selected to be comparable, featuring the same anchor identity (Luo Yonghao and his digital avatar), the same product category (carbonated beverages), and similar script structure, a single-stimulus design means that observed effects may partly reflect idiosyncratic features of the specific videos rather than anchor type per se. The two conditions also differed in promotional pricing structure, which may have influenced perceived value independently of anchor type. Future studies should use multiple matched stimuli per condition or self-produced controlled videos with identical pricing to strengthen causal inference.

Third, this study did not include dedicated measures of perceived anthropomorphism or social presence. The manipulation check data provide partial evidence that the AI anchor was perceived as possessing social-agent qualities (perceived humanness above the scale midpoint), and the theoretical framework draws on the CASA paradigm to justify treating AI anchors as social agents. However, the degree of social-agent perception may vary across AI anchor designs, and future research should incorporate validated anthropomorphism scales (e.g., Bartneck et al., 2009) to more precisely quantify this perception and to test whether anthropomorphism mediates or moderates the effects observed here.

Fourth, the product category was not experimentally varied. The present study used carbonated beverages, a low-involvement product category, which may limit generalizability to high-involvement categories where trust concerns are more salient. Future studies should manipulate both anchor type and product category to test potential interaction effects.

Fifth, the data were collected exclusively from Chinese consumers. Live-streaming commerce is highly developed in China, and cultural norms regarding trust, technology acceptance, and parasocial interaction may differ substantially from those in other cultural contexts. Cross-cultural studies would be valuable to assess the generalizability of these findings.

Sixth, the study focused on perceived intimacy and perceived responsiveness as the primary identity-based cues. While these were selected because they are the most directly observable cues in live-streaming interaction, other cues, such as perceived moral integrity, transparency, humor, or novelty, may also influence trust formation. Gao et al. (2025) have shown that novelty seeking moderates the relationship between streamer type and purchase intention, suggesting that individual difference variables beyond hedonic and utilitarian motivation deserve attention. Future studies could expand the cue set and explore longitudinal dynamics to examine whether consumer trust in AI anchors evolves as familiarity accumulates over time.

Seventh, although the study draws on the CASA paradigm and the psychology of AI literature to frame the findings, the boundaries of these frameworks in interactive commerce contexts remain to be fully mapped. Recent research has questioned whether CASA effects hold uniformly across all technology types and interaction modalities (Williams and Lim, 2024). Future research should test whether the conditional trust pathways identified here generalize to other forms of human–AI interaction, such as AI customer service agents, AI-generated content, or AI companions in social media contexts.

## 6 Conclusion

This study examined the effects of anchor type (AI versus human) on consumer trust and purchase intention in live-streaming commerce, approaching the question from a media psychology and human–machine communication perspective. The findings reveal that anchor type influences trust through two distinct identity-based cue pathways that operate asymmetrically. Human anchors primarily build trust via perceived intimacy, a relational cue whose effect is strengthened by hedonic motivation and remains relatively stable across motivational contexts. AI anchors hold a strong advantage in perceived responsiveness, a functional cue, but this advantage translates into trust only when consumers’ utilitarian motivation is high. Without the matching motivational context, AI anchors’ responsiveness advantage fails to convert into consumer trust. These findings provide a principled answer to the question of whether AI anchors can replace human anchors: they maybe not, at least not broadly. The relational pathway that human anchors command is more broadly effective, giving human anchors an overall advantage in trust formation. But AI anchors are not without value; for efficiency-oriented consumers, the functional pathway they command can be highly effective. The relationship between AI and human anchors is therefore conditional rather than substitutional. Trust formation in live-streaming commerce is a motivation-dependent, cue-matching process, and successful deployment of AI anchors requires strategic alignment between the anchor’s identity cues and the consumer’s motivational goals.

## Data Availability

The raw data supporting the conclusions of this article will be made available by the authors, without undue reservation.
